# Treatment satisfaction, adherence and behavioral assessment in patients de – escalating from natalizumab to interferon beta

**DOI:** 10.1186/1471-2377-14-38

**Published:** 2014-02-28

**Authors:** Chiara Zecca, Gianna C Riccitelli, Pasquale Calabrese, Emanuele Pravatà, Ursula Candrian, Charles RG Guttmann, Claudio Gobbi

**Affiliations:** 1 Neurocentre of Southern Switzerland, Ospedale regionale di Lugano, Lugano, Switzerland; 2 Division of Cognitive and Molecular Neuroscience, University of Basel, Petersplatz 1, Basel, 4003, Switzerland; 3 Center for Neurological Imaging, Departments of Radiology and Neurology, Brigham & Women’s Hospital, Harvard Medical School, Boston, MA, USA

**Keywords:** Adherence, Fatigue, Cognition, Quality of life, Multiple sclerosis, Natalizumab, Interferon beta-1b

## Abstract

**Background:**

De-escalating natalizumab (NTZ) to interferon beta 1b (IFN B 1B) is a possible treatment option in multiple sclerosis (MS) patients interrupting NTZ because of increased risk of progressive multifocal leukoencephalopathy (PML). The aim of this study was to evaluate satisfaction and adherence to treatment, behavioral and fatigue changes in patients switched to IFN B 1B compared to continued NTZ treatment.

**Methods:**

A 1 year, prospective, randomized, rater-blinded, parallel-group study. Nineteen relapsing remitting (RR) MS patients, randomly assigned to undergo either NTZ (n = 10) or IFN B 1B (n = 9) treatment, who had previously received NTZ for at least 12 months with disease stability and fearing or at risk for PML were included. Patients underwent behavioral and treatment assessments at baseline, after 24-week and 1 year follow-up. Behavioral assessment included measures of cognition, fatigue and quality of life. Treatment assessment included measures of satisfaction, persistence and adherence to treatment. Clinical-radiological disease activity and safety were also assessed.

**Results:**

Baseline characteristics of patients were similar between groups except for Euro Quality Visual Analogue Scale, being higher in the NTZ group (p = 0.04). Within-group comparisons at the three time points, as well as interaction analysis of treatment effect over time did not show any statistically significant differences in behavioral or treatment assessments, but a coherent trend favoring NTZ over IFN B 1B.

**Conclusions:**

De-escalating NTZ to IFN B 1B is feasible and associated with overall good patient related outcome and persistently stable behavioral measures.

## Background

Interferon beta (IFN B 1B) and glatiramer acetate (GA) are the most widely prescribed first line disease modifying drugs for relapsing remitting multiple sclerosis (RRMS). They have proven to be efficacious in reducing relapse frequency and disability progression in the short term. NTZ, a novel drug approved in Europe as second line therapy, exerts a robust anti-inflammatory activity in RRMS, but is associated with an increased risk of progressive multifocal leukoencephalopathy (PML) [1-8] which limits its prolonged use in the majority of cases. However, NTZ cessation is followed by a reactivation of disease activity [9] and no established guidelines exist on patient risk stratification and management. A few recent reports have explored the possible role of first line MS disease modifying therapies as de-escalating compounds after NTZ cessation [10-12], showing a moderate capability to limit recrudescence of disease activity. Besides efficacy, patient-reported outcomes (PRO) are crucial when addressing the selection of a specific treatment, especially in the long term. Here we present changes in treatment satisfaction and adherence, as well as in behavioral assessments including measures of cognitive functions, fatigue and quality of life after NTZ de-escalation to IFN B 1B in a small group of patients in clinical practice.

## Methods

### Ethics statement

The study protocol was approved by the local ethics committee (Comitato Etico Cantonale del Canton Ticino) and Swissmedic, and conducted in accordance with the ethical principles of the Declaration of Helsinki and the ICH-GCP Guidelines of 17 Jan. 1997 (G.U. n 191, 18 Ago 1997) and with the appropriate national regulations. Patients provided written informed consent before study enrollment.

### General study description

This is a pre-planned analysis of the study reported at ClinicalTrial.gov ID: NCT1144052, testing the employment of IFN B 1B as a de-escalating option following NTZ treatment. The design and clinico-radiological efficacy results have been previously described in detail [12].

The trial was a 1-year, prospective, controlled, randomized, rater blinded, parallel-group, monocentric pilot study that included 19 patients with RRMS according to 2005 McDonald criteria [13] from 2010 to 2011. Main inclusion criteria were age between 18 and 60, being at significant risk for (i.e. NTZ treatment duration equal to or greater than 12 months) or fear of PML, and being free of disease activity (free from relapses and disability progression for at least 6 months and no gadolinium enhancing lesions [Gd + L] on baseline [BL] MRI). Included patients were randomly assigned in a 1:1 ratio to continue monthly intravenous (i.v.) NTZ 300 mg or to de-escalate to subcutaneous (s.c.) IFN B 1B 250 μg every other day. The study was powered for MRI outcomes. Here we report the secondary and tertiary outcomes.

Complete behavioral assessment, treatment satisfaction, persistence and adherence over 1 year were evaluated in the de–escalation group and compared to continued NTZ treatment.

### Behavioral assessment

At baseline (t0), 6 months (t1) and 1 year (t2), behavioral assessment of patients included Paced Auditory Serial Addition Test, 3 sec (PASAT), Fatigue Scale for Motor and Cognitive functions (FSMC), Functional Assessment of Multiple Sclerosis (FAMS), and EuroQuol visual analogue scale (EQ-VAS).

The PASAT is a measure of sustained attention and speed of information processing [14]. This test is widely used in many cognitive research batteries for MS and is included in formal clinical outcome measures such as the MS Functional Composite Measure [15]. To avoid as much as possible a “practice effect” on the cognitive outcomes, parallel forms (form A and form B) of tests were alternatively administered at consecutive cognitive evaluations.

The FSMC is a self-administered and validated questionnaire specifically developed for MS related fatigue symptoms [16]. It consists of 20 items and allows graduation of fatigue severity, and separate evaluation of motor (10 items) and cognitive (10 items) fatigue components. Each question is rated on a five-point Likert scale. A total score greater than 43 indicates the presence of fatigue and the score increases proportionally to the severity of fatigue up to a maximum score of 100.

The FAMS consists of a generic core health-related quality of life (QoL) measure, supplemented with MS specific items [17]. A total score for overall QoL is derived by adding the scores for all subscales excluding 'additional concerns’. It ranges from 0 to 176, and a higher score reflects a better QoL [17].

The EQ-VAS is the last component of EQ-5D (EuroQuol 5 dimension), a brief standardized and generic measure of health-related QoL that provides a functional profile of an individual patient and a global health state rating [18]. EQ-VAS consists of a 10 centimetres visual analogue scale (VAS). The respondents can report their perceived health status ranging from 0 (worst imaginable health state) to 100 (best imaginable health state).

### Treatment assessment

At t1 and t2 the treatment satisfaction (TS) was quantified with TS-VAS, a 10 centimeters visual analogue scale by which respondents can report their perceived satisfaction related to therapy ranging from 0 (worst satisfaction) to 100 (best satisfaction).

In all patients the persistence on treatment and the adherence to therapy were reported. Persistence on treatment was measured as percentage of patients remaining on treatment for the whole evaluation period, and adherence to the therapy was defined as the ratio between the number of administered injections (or infusions) and the approved dosage [19,20]. Data were obtained from drug accountability at study site.

### Clinical-radiological assessments and safety

Complete clinical-radiological methods have been previously published by our group [12]. Patients were evaluated every three months with Expanded Disability Status Scale (EDSS) [21], and brain MRI [22]. Quarterly physical examination, registration of local and systemic adverse events, laboratory analysis as well as brain MRI were part of the safety follow-up. Clinical evaluation and brain MRI were performed in any case of neurological deterioration.

### Statistical analysis

Baseline demographic and clinical measures were compared between groups using the Two-sided exact Fisher Test and the Mann–Whitney Test. In each group, longitudinal generalized linear models for repeated measures were used to assess changes of neuropsychological/behavioral performance over time based on Poisson distribution. An unstructured covariance matrix was used to model the repeated measurements. We tested specific contrast: t1 *vs* t0, t2 *vs* t0, and t2 *vs* t1, and we assessed an overall time trend. Differential treatment effect over time was assessed including the group *x* time interaction term into the models. Given the small samples size we applied a correction for Type I error using for each neuropsychological end point an adjustment for multiple comparisons following the Bonferroni method. Statistical analyses were performed using the SPSS 13.0 software package (SPSS Inc., Chicago, IL, USA).

## Results

Nineteen patients were included (NTZ =10, IFN B 1B = 9).

Table 1 summarizes baseline measures related to demographics and clinical parameters that were similar between groups. At baseline, all clinical variables were similar between groups (PASAT p = 0.68, FSMC p = 0.93, FAMS p = 0.33), except for EQ-VAS measure that was lower in the IFN B 1B group (p = 0.04).

**Table 1:** Demographic and clinical characteristics of MS patients on account of treatment

	**IFN B 1B group (n = 9)**	**NTZ group (n = 10)**	**p-value**
Women/men	3/6	6 /4	0.18
Mean age, (range) [years]	39 (24–48)	43 (20–60)	0.46
Median disease duration, (range) [years]	12 (2–23)	10 (5–17)	0.71
Median NTZ infusions (range) [years]	21 (12–49)	25.5 (13–45)	0.66
Annualized relapse rate during NTZ treatment	0 (0)	0 (0–1.3)	0.50
Median EDSS (range) [years]	3.0 (1.5-3.5)	3.0 (1.5-3.5)	0.71

*Abbreviations*: *MS* multiple sclerosis; *NTZ* natalizumab; *IFN B 1B* interferon; *EDSS* expanded disability status scale.

17/19 patients completed the study: one IFN B 1B-patient withdrew consent (day 34) because she could not comply with study procedures; one NTZ-patient opted for an oral treatment (day 139). Patient (#9) switched from IFN B 1B to rescue treatment with GA at day 69 due to systemic side effects.

Table 2 summarizes the results of behavioral evaluation and treatment assessment in the two study groups at the three time points.

**Table 2:** Clinical outcomes (means ± standard deviation) of the IFN B 1B and NTZ group at the study time points

	**IFN B 1B Group**			*p value				**NTZ group**			*p value				**p values
**Behavioural assessment**	**t0 (SD)**	**t1 (SD)**	**t2 (SD)**	**t1vs t0**	**t2 vs t0**	**t2 vs t1**	**Time trend**	**t0 (SD)**	**t1 (SD)**	**t2 (SD)**	**t1 vs t0**	**t2 vs t0**	**t2 vs t1**	**Time trend**	
PASAT	44.7 (6.9)	45.1 (7.8)	49.1 (8.3)	1	0.3	-	0.2	42.0 (11.1)	45.0 (9.6)	47.5 (8.3)	0.8	0.1	-	0.1	0.8
FSMC	59.4 (17.7)	62.9 (17.6)	68.9 (12.3)	0.7	0.2	-	0.08	55.5 (19.1)	49.8 (16.5)	53.1 (13.9)	0.5	1	-	0.4	0.9
FAMS	122.4 (26.9)	116.9 (32.1)	109.2 (25.3)	1	0.2	-	0.2	133.3 (21.5)	132.7 (17.4)	133.7 (15.4)	1	1	-	0.9	0.2
EQ-VAS	64.3 (13.5)	59.2 (12.8)	59.8 (11.3)	0.9	1	-	0.5	78.5 (8.9)	71.2 (16.8)	77.3 (9.8)	0.3	1	-	0.2	0.6
TS	-	80.0 (11.1)	78.6 (10.2)	-	-	0.4	0.4	-	96.0 (6.9)	93.0 (7.3)	-	-	0.1	0.1	0.6
Adherence	-	91.7 (7.33)	90.6 (5.7)	-	-	0.6	0.6	-	100 (.00)	99.2 (2.5)	-	-	0.3	0.3	0.9

*p = longitudinal generalized linear models; **p = group *x* time interaction.

*Abbreviation*: *NTZ* natalizumab; *IFN B 1B* interferon; *EDSS* expanded disability status scale; *PASAT 3*” paced auditory serial addition test 3 sec.; *FSMC* fatigue scale for motor and cognition functions; *FAMS* functional assessment of multiple sclerosis; *EQ-VAS* euroQuol visual analogue; *TS* treatment satisfaction *SD* standard deviation.

**Figure 1:**
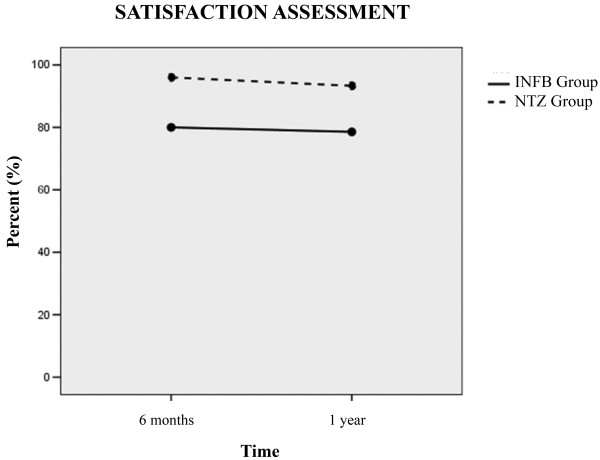
Assessment of treatment satisfaction at month six and at year 1 of study in natalizumab (NTZ group) [dotted line] and interferon (IFN B 1B group) [continuous line].

Within-group comparisons of behavioral and treatment assessments at t1 *vs* t0, t2 *vs* t0, and t2 *vs* t1 showed no significant differences (p values ranging from 0.08 to 1). The interaction analysis of treatment effect over time showed no significant changes for cognitive function (PASAT), fatigue (FSMC), and quality of life (FAMS, EQ-VAS) (p = 0.8, p = 0.9, p = 0.2, respectively), as well as treatment satisfaction (TS-VAS) (p = 0.6) (Figure 1). Nonetheless, coherent trends in favor of NTZ were observed for the majority of these outcomes. The main reasons for treatment satisfaction were “convenience of use” and “no MS illness recall” for NTZ and “optimal safety” for IFN B 1B.

Persistence on treatment was 90% (9/10) for NTZ, and 78% for IFN B 1B (7/9).

Treatment adherence over the study period was high overall and showed a similar trend between groups (p = 0.9, Table 2). Main reasons for missing IFN B 1B injections were: “forget to administer” (75%) and “concomitant fever” (9%). One patient didn’t receive one NTZ infusion because of concomitant hospitalization for gastroenteritis.

Clinical-radiological outcomes have been reported in detail in Gobbi et al. [12], and are summarized in Table 3. Briefly, a trend towards a superior efficacy of NTZ compared to IFN B 1B was found in the majority of the clinical and radiological outcome parameters.

**Table 3:** Clinical and radiological study outcomes

	**IFN B 1B**	**NTZ**	**p-value**
	**(n = 9)**	**(n = 10)**	
Number of relapses, median (range)^	3	0	0.447
- month 1 to 6	2	0 (0)	
- month 6 to 12	1	0 (0)	
Proportion of relapse free patients over the follow up period (number)°	78% (7)	100% (10)	0.206
Annualized relapse rate			
- during run-in period NTZ	0 (0)	0 (0–1.3)	0.497
EDSS			
- at month 6	3.0 (1.5-3.5)	3.0 (1.5-3.5)	0.966
- at month 12	3.5 (1.5-3.5)	3.0 (1.5-3.5)	0.315
Number of new T2 lesions, median (range) ^			
- at month 6	1.5 (0–9)	0 (0–2)	0.043
- at month 12	0 (0–12)	0 (0)	0.234
Number of Gd enhancing lesions, median (range) ^			
- at month 6	0 (0–5)	0 (0)	0.442
- at month 12	0 (0–2)	0 (0–1)	0.694

^U-Mann Whitney test; °two-sided exact Fisher test.

Adverse events were within the expected range in both groups. Skin reactions occurred in 44% of patients in the IFN B 1B group, and self-limiting infections occurred in both groups at comparable rates (33% for IFN B 1B vs 50% for NTZ, p = 0.65). None of the patients continuing NTZ developed PML symptoms or signs during the course of this study.

## Discussion and conclusions

PML risk stratification in patients undergoing NTZ treatment can be performed based on treatment duration, history of previous immunosuppression and, more recently, on serology for JCV [3-6]. The decision to discontinue NTZ may be derived from an unfavorable risk/benefit ratio calculated on the basis of probabilistic clinical reasoning [23]. Patients may also independently interrupt NTZ for fear of developing PML. Overall, in clinical practice up to 60% of patients with JCV seropositivity discontinue treatment [23]. Recently, some studies have been conducted that address the efficacy of first line compounds for MS after NTZ discontinuation, showing a partial efficacy for glatiramer acetate and IFN B 1B [10,11]. Adherence to treatment and how treatment might change the quality of life are key issues of patient outcomes in chronic conditions where long-term treatments are required. The awareness of this concern has grown among neurologists to such an extent that patient-reported outcomes (PRO) are nowadays included even in larger MS clinical trials (ClinicalTrials.gov ID NCT00751881). To the best of our knowledge, no previous report on satisfaction of patients de-escalating from NTZ to a first line, injectable disease modifying agent for MS is available.

Behavioral measures as well as treatment satisfaction and adherence in the IFN B 1B group were comparable to those in the group with continued NTZ treatment. Overall, NTZ was coherently favored in the majority of the outcomes, even though this trend did not reach statistical significance in any of the measures taken alone. A larger study would be necessary to validate this finding.

We evaluated changes in cognitive performance in the two groups over time. At study entry both groups obtained high PASAT scores suggesting preserved orbito-frontal pathways involved in working memory maintenance, manipulation and monitoring processes. We then found that both treatments were associated with non-significant improvement of cognitive performances over time. Our results are in line with Fischer’s findings [24], showing a positive effect of IFN B 1B on cognitive domains investigated also with the PASAT test in a wide sample of RRMS patients. This result, however, differs from previous studies [25], which found NTZ to be more effective than IFN B 1B in reducing cognitive deterioration. Several factors are likely to contribute to explain discrepancies between our and previous findings, including limited cognitive impairment at baseline, short duration of follow-up, and tests used for the assessment of cognitive performance. A “practice effect” might have also contributed, however the use of parallel forms of PASAT should have mitigated it at least to some extent.

Fatigue assessment also showed a trend favoring NTZ after 6 and 12 months of NTZ de-escalation to IFN B1 B, and compared to continued NTZ treatment. The role of interferons in MS fatigue is controversial. Some authors found interferons to aggravate fatigue [26,27], while others showed interferons to be irrelevant to fatigue [28,29]. On the other hand, escalation to NTZ has been shown to be associated with a perceived reduction of fatigue [30,31]. In our study, fatigue might have been influenced by disease recurrence in some patients in the IFN B 1B arm.

QoL on IFN B 1B was comparable to previous treatment with NTZ and to continued NTZ therapy at 6 and 12 months’ time points. This result may be related to the substantial satisfaction with the treatment expressed by patients on IFN B 1B, which was similar to that of the NTZ group (p = 0.6). Interestingly, the main reasons for treatment satisfaction were “convenience of use” and “no MS illness recall” for NTZ and “optimal safety” for IFN B 1B. Treatment safety remains a crucial concern for patients undergoing NTZ treatment, and possibly counterbalances the disadvantage of IFN B 1B administration route and side effects, when assessing overall quality of life. Indeed, in our clinical practice the fear of PML is sometimes followed by development of disabling mental distress ultimately associated to a significant worsening of quality of life. Similar findings were also reported by van Rossum et al., showing a correlation between unsafe feelings related to the risk of PML, and anxiety in MS patients treated with NTZ [32].

Adherence to treatment reached values above 90% in the IFN B 1B group at each time point, which is greater than the mean value described in the literature [33-35]. Also, a relatively high proportion of patients persisted on treatment in both groups (IFN B 1B nearly 80% vs NTZ 90%), similarly to the findings from larger trials that evaluated MS therapies over similar timeframes [36,37]. An association between adherence to disease modifying agents and MS patients’ clinical outcome has been outlined in several works, with lower risks of relapses, hospitalizations and reduced quality of life in patients with regular intake of medications [35,38].

The main limitation of our study is represented by the small sample size. High retention and adherence to IFN B 1B treatment may reflect a bias of a patient sub-population that is willing to participate in a clinical study. Moreover, our sample of patients is fully representative of the MS patient population treated with NTZ in southern Switzerland as all patients receiving NTZ in this region are treated at our site, and clinical and radiological characteristics of the patients included in the study were similar to remaining NTZ treated patients at our site (data not shown). Moreover, we postulate that our study population is well representative of a typical NTZ treated population as included patients experienced over 90% reduction of annualized relapse rate under NTZ treatment compared to the two years before NTZ initiation, which is in line with the known efficacy of NTZ in active MS patients [39,40]. A second limitation of this sub-analysis is represented by the lack of specific scales for assessment of mood disorders, even though the FAMS questionnaire includes items related to physiological wellness. In addition, cognitive outcome should be considered with caution since we evaluated only the most frequent cognitive dysfunctions in MS (working memory, sustained attention, and information processing speed) using a single test (PASAT) as measure of cognitive impairment. Albeit, this test is a validated instrument widespread applied as screening test in MS clinical research. Finally, the study lacks a placebo arm, which was avoided for ethical reasons. These limitations may be partially mitigated by the randomized, prospective design with quarterly MRI monitoring.

In conclusion, our study suggests that de-escalation to IFN B 1B at NTZ cessation is feasible, well accepted and tolerated by the majority of patients, and seems to be associated with substantial stability of cognitive and fatigue parameters.

## Competing interests

C. Zecca has received personal compensation from Teva, Meck Serono, Biogen Idec, Bayer Scering, Novartis. G.C. Riccitelli has nothing to disclose. P. Calabrese has nothing to disclose. E. Pravatà has nothing to disclose. U. Candrian has nothing to disclose. C.R.G. Guttman has received personal compensation from Tibotec/Johnson & Johnson and was the recipient of a research grant from Teva Neuroscience, within the last 3 years. C. Gobbi has received personal compensation from Teva, Merck Serono, Biogen Idec, Bayer Schering, Novartis.

## Authors’ contributions

CZ has contributed to the design/conceptualization of the study, analysis/interpretation of the data, and drafting/revising the manuscript for intellectual content. GCR has contributed to the design/conceptualization of the study, result collection and the analysis/interpretation of the data, and revising of the manuscript for intellectual content. PC has contributed to the design/conceptualization of the study and revising the manuscript for intellectual content. EP has contributed to the analysis/interpretation of the MRI data, and revising of the manuscript for intellectual content. UC has contributed to the design/conceptualization of the study and revising the manuscript for intellectual content. CRGG has contributed to the design/conceptualization of the MRI component of the study and the analysis/interpretation of the data, and revising of the manuscript for intellectual content. CG has contributed to the design/conceptualization of the study, analysis/interpretation of the data, and drafting/revising the manuscript for intellectual content. All authors read and approved the final manuscript.

## Pre-publication history

The pre-publication history for this paper can be accessed here:

http://www.biomedcentral.com/1471-2377/14/38/prepub
